# Supplementary material for: Genetic determinants of micronucleus formation in vivo
Source: Nature. 2024 Feb 14;627(8002):130–6. doi: 10.1038/s41586-023-07009-0 (PMC10917660; doi:10.1038/s41586-023-07009-0)
Supplement: Supplementary file 1 — Supplementary Figs. 1–4, legends for Supplementary Tables 1–7, Supplementary Discussion and Supplementary References. [file 41586_2023_7009_MOESM1_ESM.pdf]

nature portfolio

https://doi.org/10.1038/s41586-023-07009-0

Supplementary information

Genetic determinants of micronucleus formation in vivo

In the format provided by the authors and unedited

Genetic determinants of micronucleus formation *in vivo*

Adams, D.J., Barlas, B., McIntyre, R.E., Salguero, I., van der Weyden, L., Barros A., Vicente, J.R., Karimpour N., Haider A., Ranzani, M., Turner, G., Thompson, N.A., Harle V., Olvera-León R, Robles-Espinoza CD, Speak, A.O., Geisler N., Weninger, W. J., Geyer, S. H., Hewinson, J., Karp, N.A., The Sanger Mouse Genetics Project, Fu, B., Yang, F.[1], Kozik Z., Choudhary J., Yu L., van Ruiten, M.S., Rowland, B.D., Lelliott C.J., del Castillo Velasco-Herrera M., Verstraten, R., Bruckner, L., Henssen, A. G., Rooimans, M.A., de Lange, J., Mohun, T. J., Arends, M. J., Kentistou, K.A., Coelho, P.A., Zhao, Y., Zecchini, H., Perry, J R. B., Jackson, S. P. and Balmus, G.

Correspondence: gb318@cam.ac.uk and da1@sanger.ac.uk

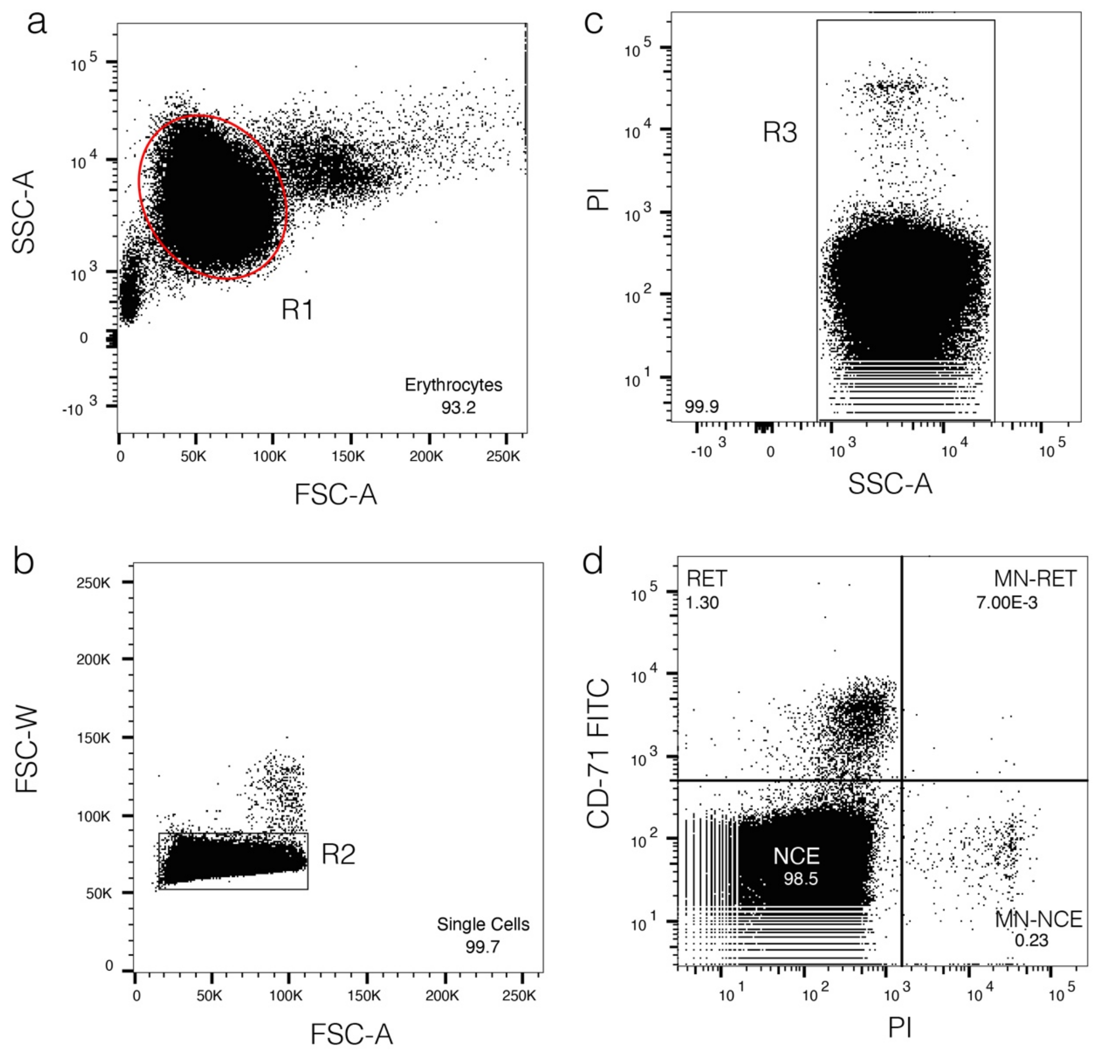

Supplementary Figure 1. Gating strategy to quantify micronucleated red blood cells by flow cytometry. (a) Red blood cells are distinctly separated from other cells using a specific region gate R1, defined on a log scale for side scatter area (SSC-A-log) and a linear scale for forward scatter area (FSC-A-lin). (b) Individual cells are singled out using region gate R2, based on forward scatter width (FSC-W) in relation to FSC-A-lin. (c) Cells exhibiting autofluorescence are excluded through a gating process involving propidium iodide (PI) and SSC-A. (d) The cells sorted through gates R1, R2, and R3 are then analyzed based on their fluorescence intensity in the FITC channel (CD71-FITC) and the PI channel. A quadrant gate is established to categorize the cells into four distinct groups: normochromatic erythrocytes (NCEs) identified as CD71−/PI−; reticulocytes (RETs) as CD71+/PI−; micronucleated reticulocytes (MN-RETs) as CD71+/PI+; and micronucleated normochromatic erythrocytes (MN-NCEs)

as CD71−/PI+. For a detailed protocol see Balmus et al. (2015) Nature protocols 10, 205-215[1].

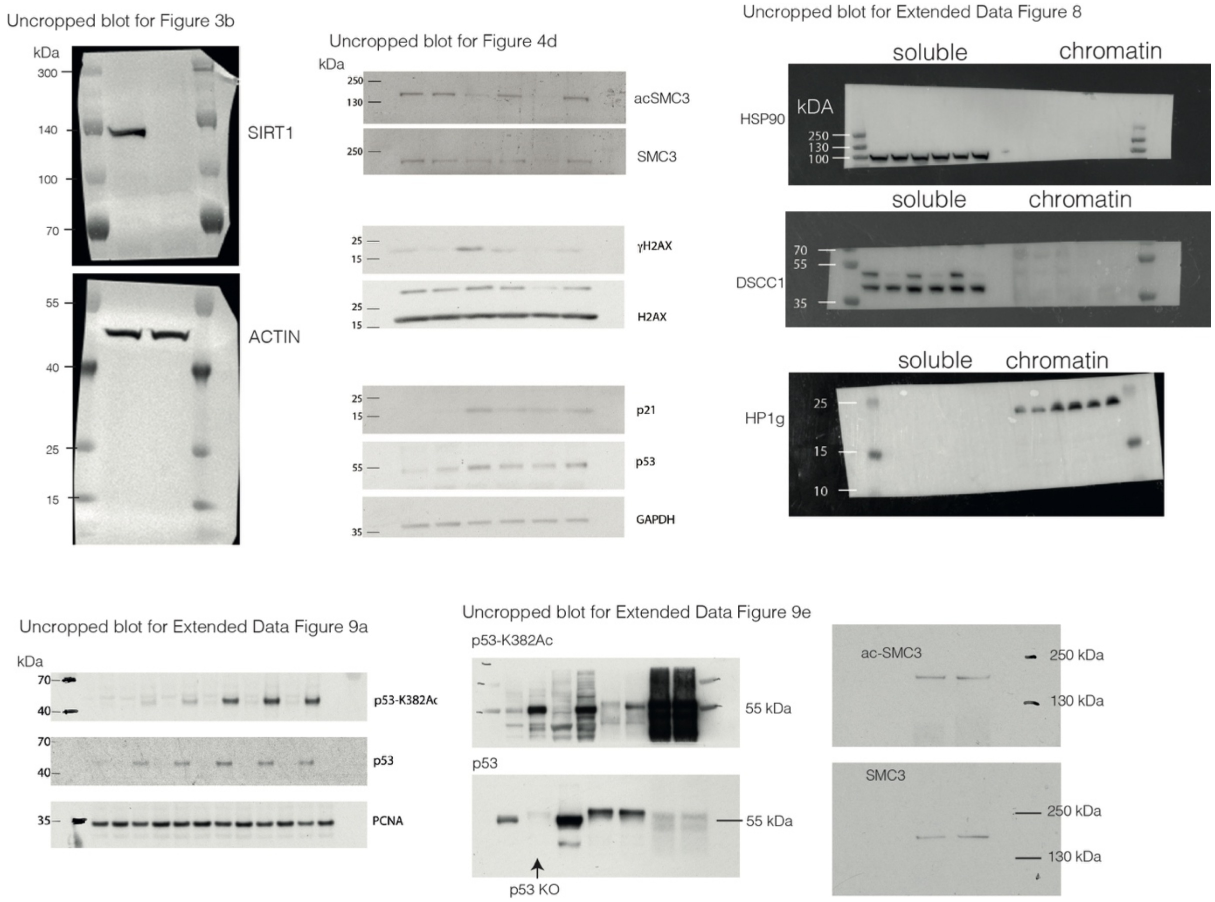

Supplementary Figure 2. Uncropped images for the western blots used in this paper.

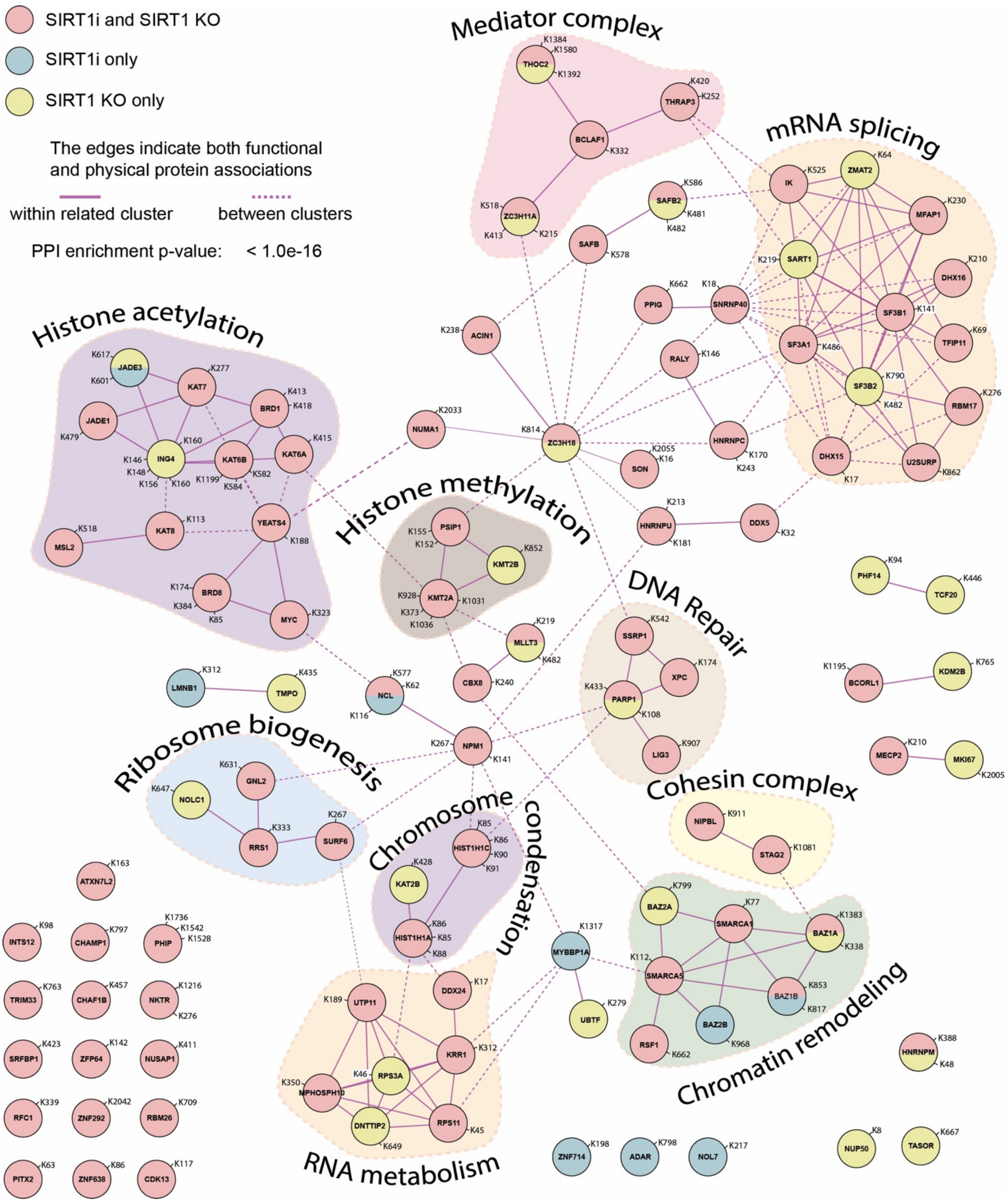

Supplementary Figure 3. Determination of the SIRT1i specificity in HEK293 and HEK293 *SIRT1* KO cells. Interactome analysis using STRING v12[2] of the chromatin acetylome mass-spectrometry (MS) data. Represented are the acetylated proteins that we define as "TRUE" based on an adjusted P-value <0.05 and a Log2 fold change >1 (Students' two-tailed T-test). Data was generated from three biological replicates. The STRING interaction network (enrichment P<1.0E-16) is based on an active interaction source with only experiments considered at a medium confidence score

(0.400) and was computed using the Hypergeometric test with Benjamini Hochberg correction for multiple testing[2,3]. For each protein the lysine acetylation sites are indicated. The red circles are SIRT1 substrates that overlap between SIRT1i treated and *SIRT1* KO cells. The blue circles are proteins present only in the SIRT1i treated condition. Yellow circles indicate proteins present only in the HEK293 *SIRT1* KO cells. Circles with two colours represent different acetylation sites in the respective categories. The edges indicate both functional and physical protein associations and common cluster names are indicated. See Source Data for Supplementary Figures 2-3.

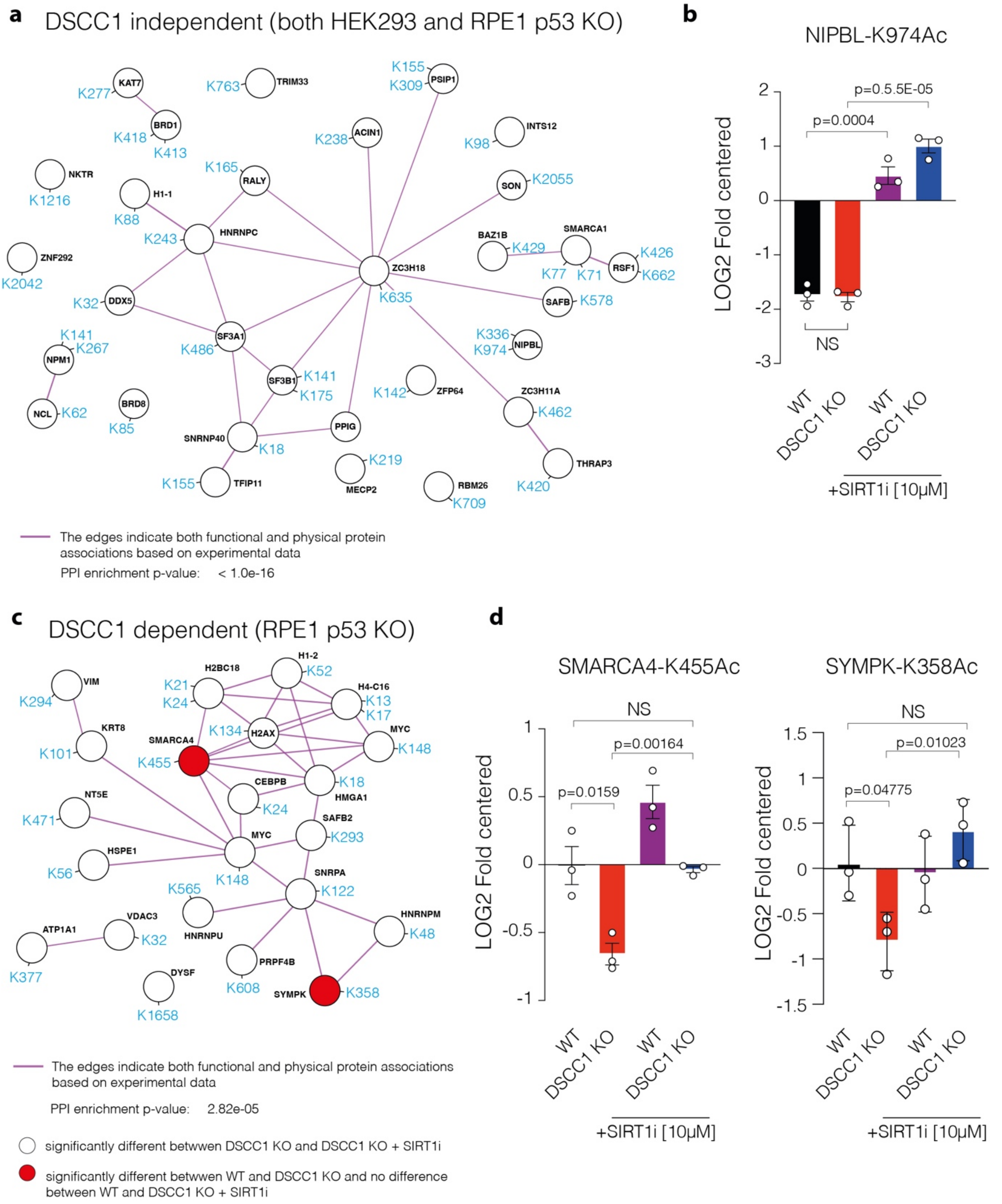

Supplementary Figure 4. Determination of the SIRT1i specificity in relationship to *DSCC1* KO in RPE-1 *p53* KO cells. (a) Interactome analysis using STRING v.12[2] of the chromatin acetylome mass-spectrometry data representing an overlap of SIRT1 substrates between HEK293 and RPE-1 *p53 KO* cells. Represented are the acetylated proteins that we define as "TRUE" based on an adjusted *P* value <0.05 and a Log2 fold change >1 (Students' two-tailed t-test). n=3 biological replicates. The

STRING interaction network (enrichment *P*<1.0E-16) is based on an active interaction source with only experiments considered at a medium confidence score (0.150). and was computed using the Hypergeometric test with Benjamini Hochberg correction for multiple testing[2,3]. For each protein, the lysine acetylation sites are indicated. **(b)** Bar graph depicting the abundance of NIPBL K974 acetylation in RPE-1 *p53 KO* cells as compared to RPE-1 *p53 KO* and *DSCC1* KO cells in presence or absence of SIRT1i. Statistical analysis is Students' two-tailed t-test. Error bars are s.d. n=3 biological replicates. **(c)** Interactome analysis using STRING v.12[2] of the chromatin acetylome mass-spectrometry data presented in Source Data and representing the differences between *p53* KO *DSCC1* KO cells and *p53* KO *DSCC1* KO cells treated with SIRT1i. Represented are the acetylated proteins that we define as "TRUE" based on an adjusted *P*-value <0.05 and a Log2 fold change >1 (Students' two-tailed t-test). n=3 biological replicates. The STRING interaction network (enrichment *P*<2.82E-05) is based on an active interaction source with only experiments considered at a medium confidence score (0.150), and was computed using the Hypergeometric test with Benjamini Hochberg correction for multiple testing[2,3]. For each protein, the lysine acetylation sites are indicated. The open circles, proteins with unique acetylation sites, are SIRT1 substrates that are different between *DSCC1* KO and *DSCC1* KO + SIRT1i but are not changed in the WT vs. *DSCC1* KO comparison. The red circles, K358-SYMPK and K455-SMARCA4, are SIRT1 substrates that are different in the WT vs. *DSCC1* KO comparison and that are rebalanced to WT-like stage upon SIRT1i treatment. **(d)** Bar graph depicting the abundance of SMARCA4-K455 and SYMPK-K358 acetylation in RPE-1 *p53* KO cells as compared to RPE-1 *p53* KO *DSCC1* KO cells in the presence or absence of SIRT1i showing a rebalancing of acetylation levels. Statistical analysis is Student's two-tailed t-test. n=3 biological replicates. Error bars are s.d.

Supplementary Table 1. Full micronucleus dataset and statistics.

Supplementary Table 2. Loss of Y (LOY) genome wide association studies (GWAS) links mouse micronucleus (MN) genes to human orthologues. Top: Gene-set enrichment analysis for MN genes within the various Tiers. N human genes indicates the number of MN candidate genes identifies within each Tier and N human genes with variants indicates the number of said genes, with data available to be tested. Cross-species mapping was performed with Ensembl. P indicates the *P*-value for the gene-level association between variants within genes belonging to each tier or group of tiers and variation in LOY). Bottom: Overlap between identified MN genes in mice and human datasets. Genes within Tiers 1, 2 or 3 which showed any level of association towards the human LOY phenotype, across the 4 analyses (Extended Data Fig. 2). (gene; gene name, n# analyses; number of analyses out of 4, which highlighted corresponding gene, chr; chromosome where human gene is located, start; start site of gene, end; end site of gene, snp; genome-wide significant signal which is proximal to gene, pos; chromosomal position for signal on hg19, a1; effect allele of signal; a0; alternate allele, freq1; frequency of the effect allele, beta1; effect size estimate per copy of the effect allele on LOY, se; standard error of beta1, p; *P*-value for the association between snp and LOY, n; overall sample size for the LOY GWAS; magma_cv_p; *P*-value from the gene-level coding variant MAGMA test, magma_cv_q; FDR-corrected magma_cv_p, eqtl_p_SMR; *P*-value for the SMR test, eqtl_q_SMR; FDR corrected eqtl_p_SMR, eqtl_p_HEIDI; *P*-value for the HEIDI test, eqtl_smr_SNP_a0_a1; top SMR associated SNP with coordinates on hg19, eqtl_smr_direction; comparative direction seen between the eQTL and LOY GWAS data, all repeated for the pQTL analysis).

Supplementary Table 3. Overlap of +MN genes with other human datasets. Overlap between identified +MN genes in mice and human datasets.

Supplementary Table 4. Human disease associations for *DSCC1* gene. Top: DSCC1 lookup in targeted human phenotypes. Human-equivalent phenotype to the ones seen in the *Dscc1*$^{-/-}$ mouse were interrogated for common- and rare-level variant associations with *DSCC1.* (trait; tested human phenotype, chr; chromosome where *DSCC1* is located, start; start site of DSCC1, stop; end site of DSCC1, nsnps; available

SNPs between start and stop, nparam; SNPs used in association, N; sample size of GWAS, zstat; z-statistic for the gene-level MAGMA association, MAGMA_P; *P*-value for the gene-level MAGMA association, SMR-HEIDI statistics as in Supplementary Table 1). Bottom: Lookup of common variant associations within *DSCC1* via the Open Targets Genetics platform[4]. Associations with a *P* value $<5x10^{-8}$ are displayed.

Supplementary Table 5. Overlap of mouse and human phenotypes.

Supplementary Table 6. siRNAs and gRNA used in this study.

Supplementary Table 7. HU titrations.

Supplementary Discussion

Rescue of *DSCC1* loss

In this study, we explored the intricate mechanisms underlying the suppression of *DSCC1* loss-of-function cellular phenotypes by genome-wide CRISPR-Cas9 genetic screening. We show that silencing/loss of *TGFBR2, CARS, KIF25A* and *SIRT1* can rescue the cell proliferation defect associated with *DSCC1* loss (Figure 3). It was previously shown in cells that DSCC1 deficiency reduces ESCO1-dependent SMC3-Ac, causing the WAPL/PDS5A complex to hyper-engage and, subsequently, replication forks to slow and stall, leading to cell death[5]. Because gRNAs against *WAPL* were not present in our library and to clarify the role of *PDS5A* that was not recovered as a suppressor in our screen, we also tested if the depletion of these genes could rescue the DSCC1-dependent proliferation defect. We show that loss of *WAPL* but, consistent with our screen, not *PDS5A* can partially restore cellular fitness in *DSCC1*-deficient cells (Supplementary Figure 8). Interestingly, however, we saw that WAPL and PDS5A disruption/loss could rescue the increase in MN formation (Supplementary Figure 8) seen in *siDSCC1*-treated cells. These data suggest that PDS5A plays an additional, WAPL-independent, role in regulating cell fitness. Indeed, PDS5A also plays a role in DNA damage repair at DNA double-break sites[6], and recent evidence suggests that PDS5A functions in genome folding by modulating the length of loops and architectural stripes[7], a role that is WAPL-independent.

SIRT1 and the rescue cohesin phenotypes

Our paper shows that SIRT1 inhibition can restore cellular fitness and SMC3 acetylation. SMC3 acetylation during the S phase is believed to stabilise the SMC ring and ensure the maintenance of sister chromatid cohesion. The model is one in which RFC^{Ctf18} loads ESCO1 onto DNA, leading to SMC3 acetylation, a process counteracted by the HDAC8 deacetylase. Because SIRT1 is a deacetylase, we first tested if SIRT1 can directly deacetylate SMC3 and showed that, in non-stressed conditions, this is not the case. Because p53 can rescue the lethality associated with *DSCC1* KO[8] and p53 is a reported SIRT1 substrate[9], we also tested to see if the phenotypes we saw were due to p53 acetylation. We show that although p53 loss can

rescue the *DSCC1* KO loss-of-fitness phenotype, it cannot rescue the cohesion defect, but SIRT1 inhibition can in this p53 KO background. This suggests that the rescue of DSCC1 loss by SIRT1 inhibition is independent of p53.

To better understand the SIRT1-cohesion pathway connection and given the diverse roles of SIRT1, we undertook a pan-acetylation analysis via mass spectrometry and identified several proteins that showed SIRT1-dependent acetylation of which NIPBL, SYMPK and SMARCA4 stood out.

NIPBL is an essential gene and one of the most mutated CdLS genes[10]. It forms a heterodimer with MAU2, which protects NIPBL from degradation[11]. During S-phase, the NIPBL-MAU2 heterodimer is responsible for cohesion loading on chromatin, determining cohesion establishment[12]. Interestingly, while NIPBL is essential in human cells and mice, *Mau2* KO mice are viable and showed no increase in MN formation in our screen or in human cells treated with hydroxyurea to induce MN accumulation (Supplementary Table 1 and Extended data 8). Collectively these data suggest that loss of MAU2 alone is not sufficient to destabilise sister chromatid cohesion to the extent that leads to micronucleation. It has also been proposed that NIPBL can activate the cohesin ATPase to fuel loop extrusion and perhaps counteract PDS5-WAPL-mediated release[13,14]. In our experiments, while NIPBL acetylation is not affected by DSCC1 loss, upon SIRT1i, it is significantly acetylated in both WT and *DSCC1* KO cells. As far as we know, the impact of SIRT1 on NIPBL acetylation hasn't been demonstrated before, and there are no functional data available on K974-Ac, which is the most strongly modified residue in our dataset; however, we do know that this residue is a reliable site of acetylation, as it has been reported in curated mass-spectrometry databases[15]. Thus, further research will be required to determine how the NIPBL-SIRT1 connection affects cohesion.

Symplekin (SYMPK) was first identified as a protein located at tight junctions and was later found to be a part of nuclear RNA speckles[16]. It forms a complex with the cleavage and polyadenylation specificity factor (CPSF) and the cleavage stimulation factor (CstF), essential for both polyadenylation and histone cleavage processing[16,17]. It has also been reported to specify mitotic fidelity by supporting microtubule dynamics, which is critical for normal spindle formation[18]. Interestingly, recent evidence suggests that nuclear RNA speckles associate with chromatin via SYMPK, organising the

chromatin-nuclear speckles' ground state. This process is controlled by the cohesion-SYMPK interaction, as in CdLS cells (*RAD21*- Ser198Argfs*6 allele); this process is disrupted because SYMPK loses its interaction with chromatin upon cohesion disruption[19]. It is worth noting that in our studies, SYMPK-K358Ac is disrupted in *DSCC1* KO cells, and SIRT1i rectifies this imbalance. Therefore, it will be crucial to understand how DSCC1 affects this process and how K358 deacetylation by SIRT1 contributes.

SMARCA4, also known as BRG1, is a crucial component of the SWI/SNF complex and serves as its engine (ATPase) and is required for the complex to function[20,21]. It has been reported that SIRT1 can deacetylate SMARCA4 at sites of double-strand breaks, and this interaction directly stimulates its ATPase activity to relax chromatin and promote homologous recombination[22]. Interestingly, SMARCA4 loss allows for recovery of fitness caused by aneuploidy events[23]. Our study shows that in the context of a cohesion defect, SIRT1 deacetylates SMARCA4 at K455. The only modification that we found reported for K455 is ubiquitylation[24,25]. Thus, it is plausible that SIRT1 deacetylation of K455-Ac could allow its ubiquitylation, thus controlling its stability, a phenomenon seen in other instances[26,27]. This could allow for a rescue of fitness upon chromosomal instability events[23].

Summary

Overall, our data is consistent with previous literature that shows that DSCC1 loss leads to defective SMC3 acetylation and cohesion deficiency that slows down the replication fork[1] and allows for the accumulation of genomic instability that eventually leads to death via a p53-dependent pathway. SIRT1 inhibition can reverse SMC3 acetylation deficiency and rescue the associated cohesion defect, most likely indirectly. In support of this is the observation is that SIRT1 inhibition can rescue the micronucleus defects associated with SMC3 loss and defects caused by loss of CTCF and RAD21 (Extended Data Figure 8d). Cohesion dysregulation has been shown to lead to increased DNA damage following the S phase, lagging chromosomes, and micronuclei[28,29]. While we cannot exclude that failure to biorientate the mitotic spindle contributes, we speculate that SMC3-dependent cohesion imbalance due to DSCC1 loss creates an environment permissive of genome folding defects, nuclear RNA

speckle instability, and replication fork stalling that eventually can lead to double-strand breaks that fail to be repaired causing mitotic catastrophe; SIRT1 can bypass the requirement for DSCC1 by acting at multiple levels to improve genome stability. As cohesion dysregulation starts to be recognised as a player not only in CdLS but also other degenerative disorders such as Alzheimer's Disease[26], our observations on the role of SIRT1 illuminate exciting new avenues of investigation.

References

1. Balmus, G. *et al.* A high-throughput in vivo micronucleus assay for genome instability screening in mice. *Nat Protoc* **10**, 205–215 (2015).

2. Szklarczyk, D. *et al.* The STRING database in 2021: customizable protein–protein networks, and functional characterization of user-uploaded gene/measurement sets. *Nucleic Acids Res* **49**, D605–D612 (2020).

3. Franceschini, A. *et al.* STRING v9.1: protein-protein interaction networks, with increased coverage and integration. *Nucleic Acids Res.* **41**, D808–D815 (2013).

4. Ghoussaini, M. *et al.* Open Targets Genetics: systematic identification of trait-associated genes using large-scale genetics and functional genomics. *Nucleic Acids Res* **49**, D1311–D1320 (2021).

5. Terret, M.-E., Sherwood, R., Rahman, S., Qin, J. & Jallepalli, P. V. Cohesin acetylation speeds the replication fork. *Nature* **462**, 231–234 (2009).

6. Su, P.-R. *et al.* Microscopy-based single-cell proteomic profiling reveals heterogeneity in DNA damage response dynamics. *Cell Rep. Methods* **2**, 100237 (2022).

7. Ruiten, M. S. van *et al.* The cohesin acetylation cycle controls chromatin loop length through a PDS5A brake mechanism. *Nat. Struct. Mol. Biol.* **29**, 586–591 (2022).

8. Schie, J. J. van *et al.* MMS22L-TONSL functions in sister chromatid cohesion in a pathway parallel to DSCC1-RFC. *Life Sci. Alliance* **6**, e202201596 (2022).

9. Vaziri, H. *et al.* hSIR2SIRT1 Functions as an NAD-Dependent p53 Deacetylase. *Cell* **107**, 149–159 (2001).

10. Tonkin, E. T., Wang, T.-J., Lisgo, S., Bamshad, M. J. & Strachan, T. NIPBL, encoding a homolog of fungal Scc2-type sister chromatid cohesion proteins and fly Nipped-B, is mutated in Cornelia de Lange syndrome. *Nat. Genet.* **36**, 636–641 (2004).

11. Chao, W. C. H. *et al.* Structural Studies Reveal the Functional Modularity of the Scc2-Scc4 Cohesin Loader. *Cell Rep.* **12**, 719–725 (2015).

12. Murayama, Y. & Uhlmann, F. Biochemical reconstitution of topological DNA binding by the cohesin ring. *Nature* **505**, 367–371 (2014).

13. Davidson, I. F. & Peters, J.-M. Genome folding through loop extrusion by SMC complexes. *Nat. Rev. Mol. Cell Biol.* **22**, 445–464 (2021).

14. Alonso-Gil, D. & Losada, A. NIPBL and cohesin: new take on a classic tale. *Trends Cell Biol.* **33**, 860–871 (2023).

15. Hornbeck, P. V. *et al.* PhosphoSitePlus, 2014: mutations, PTMs and recalibrations. *Nucleic Acids Res.* **43**, D512–D520 (2015).

16. Takagaki, Y. & Manley, J. L. Complex Protein Interactions within the Human Polyadenylation Machinery Identify a Novel Component. *Mol. Cell. Biol.* **20**, 1515–1525 (2000).

17. Sullivan, K. D., Steiniger, M. & Marzluff, W. F. A Core Complex of CPSF73, CPSF100, and Symplekin May Form Two Different Cleavage Factors for Processing of Poly(A) and Histone mRNAs. *Mol. Cell* **34**, 322–332 (2009).

18. Cappell, K. M., Larson, B., Sciaky, N. & Whitehurst, A. W. Symplekin Specifies Mitotic Fidelity by Supporting Microtubule Dynamics. *Mol. Cell. Biol.* **30**, 5135–5144 (2010).

19. Yu, R. *et al.* CTCF/cohesin organize the ground state of chromatin-nuclear speckle association. *bioRxiv* 2023.07.22.550178 (2023) doi:10.1101/2023.07.22.550178.

20. Mittal, P. & Roberts, C. W. M. The SWI/SNF complex in cancer — biology, biomarkers and therapy. *Nat. Rev. Clin. Oncol.* **17**, 435–448 (2020).

21. Centore, R. C., Sandoval, G. J., Soares, L. M. M., Kadoch, C. & Chan, H. M. Mammalian SWI/SNF Chromatin Remodeling Complexes: Emerging Mechanisms and Therapeutic Strategies. *Trends Genet.* **36**, 936–950 (2020).

22. Chen, Y. *et al.* A PARP1–BRG1–SIRT1 axis promotes HR repair by reducing nucleosome density at DNA damage sites. *Nucleic Acids Res.* **47**, 8563–8580 (2019).

23. Schiavoni, F. *et al.* Aneuploidy tolerance caused by BRG1 loss allows chromosome gains and recovery of fitness. *Nat. Commun.* **13**, 1731 (2022).

24. Boeing, S. *et al.* Multiomic Analysis of the UV-Induced DNA Damage Response. *Cell Rep.* **15**, 1597–1610 (2016).

25. Akimov, V. *et al.* UbiSite approach for comprehensive mapping of lysine and N-terminal ubiquitination sites. *Nat. Struct. Mol. Biol.* **25**, 631–640 (2018).

26. Shimizu, K. *et al.* Interplay between protein acetylation and ubiquitination controls MCL1 protein stability. *Cell Rep.* **37**, 109988 (2021).

27. Venne, A. S., Kollipara, L. & Zahedi, R. P. The next level of complexity: Crosstalk of posttranslational modifications. *PROTEOMICS* **14**, 513–524 (2014).

28. Haarhuis, J. H. I. *et al.* The Cohesin Release Factor WAPL Restricts Chromatin Loop Extension. *Cell* **169**, 693-707.e14 (2017).

29. Yueh, W.-T., Singh, V. P. & Gerton, J. L. Maternal Smc3 protects the integrity of the zygotic genome through DNA replication and mitosis. *Development* **148**, dev199800 (2021).
