# Supplementary material for: Genetic determinants of micronucleus formation in vivo
Source: Nature. 2024 Feb 14;627(8002):130–6. doi: 10.1038/s41586-023-07009-0 (PMC10917660; doi:10.1038/s41586-023-07009-0)
Supplement: Supplementary file 2 — Reporting Summary [file 41586_2023_7009_MOESM2_ESM.pdf]

nature portfolio

Corresponding author(s): David Adams and Gabriel Balmus

Last updated by author(s): NOV_2023

Reporting Summary

Nature Portfolio wishes to improve the reproducibility of the work that we publish. This form provides structure for consistency and transparency in reporting. For further information on Nature Portfolio policies, see our Editorial Policies and the Editorial Policy Checklist.

Statistics

For all statistical analyses, confirm that the following items are present in the figure legend, table legend, main text, or Methods section.

n/a	Confirmed	
☐	☒	The exact sample size (n) for each experimental group/condition, given as a discrete number and unit of measurement
☐	☒	A statement on whether measurements were taken from distinct samples or whether the same sample was measured repeatedly
☐	☒	The statistical test(s) used AND whether they are one- or two-sided<br>*Only common tests should be described solely by name; describe more complex techniques in the Methods section.*
☐	☒	A description of all covariates tested
☐	☒	A description of any assumptions or corrections, such as tests of normality and adjustment for multiple comparisons
☐	☒	A full description of the statistical parameters including central tendency (e.g. means) or other basic estimates (e.g. regression coefficient) AND variation (e.g. standard deviation) or associated estimates of uncertainty (e.g. confidence intervals)
☐	☒	For null hypothesis testing, the test statistic (e.g. F, t, r) with confidence intervals, effect sizes, degrees of freedom and P value noted<br>*Give P values as exact values whenever suitable.*
☒	☐	For Bayesian analysis, information on the choice of priors and Markov chain Monte Carlo settings
☐	☒	For hierarchical and complex designs, identification of the appropriate level for tests and full reporting of outcomes
☐	☒	Estimates of effect sizes (e.g. Cohen's d, Pearson's r), indicating how they were calculated

Our web collection on statistics for biologists contains articles on many of the points above.

Software and code

Policy information about availability of computer code

Data collection	No commercial software of this kind was used.
Data analysis	As stated in the manuscript we used Prism software for statistical analysis (Version 9.1.0 & 10.1). Analysis of the screen results used software written in R (glmmTMB, version: 1.0.1). https://github.com/glmmTMB/glmmTMB. R V4 was used. FlowJo V7-10 was used.

For manuscripts utilizing custom algorithms or software that are central to the research but not yet described in published literature, software must be made available to editors and reviewers. We strongly encourage code deposition in a community repository (e.g. GitHub). See the Nature Portfolio guidelines for submitting code & software for further information.

Data

Policy information about availability of data

All manuscripts must include a data availability statement. This statement should provide the following information, where applicable:

- Accession codes, unique identifiers, or web links for publicly available datasets
- A description of any restrictions on data availability
- For clinical datasets or third party data, please ensure that the statement adheres to our policy

Data Availability Statement
The CRISPR screen data have been deposited to the European Nucleotide Archive with accession number ERP105493. The mass spectrometry proteomics data have been deposited to the ProteomeXchange Consortium via the PRIDE partner repository with the dataset identifiers, PXD034902, PXD030499 and PXD045110. All other data is available in the Supplementary or Source Data of the paper.

International Mouse Phenotyping Consortium Database release V19 was used. This is available via the IMPC website (www.mousephenotype.org) and also via the github page for the project.

Field-specific reporting

Please select the one below that is the best fit for your research. If you are not sure, read the appropriate sections before making your selection.

☒ Life sciences ☐ Behavioural & social sciences ☐ Ecological, evolutionary & environmental sciences

For a reference copy of the document with all sections, see nature.com/documents/nr-reporting-summary-flat.pdf

Life sciences study design

All studies must disclose on these points even when the disclosure is negative.

Sample size	Statistics and reproducibility<br>Prism (version 9.1.0 & 10.1, GraphPad) were used to perform the statistical analysis, unless otherwise described. All statistical details are provided in the Figure legends. Significance is expressed as P values (NS, not significant; P>0.5; *P<0.05, **P<0.01, ***P<0.001 and ****P< 0.0001).<br>n>3; all data-points presented in the figures.<br><br>For most experiments we had no a priori way of determining sample size so we used empirical evidence/knowledge to estimate the effect size/biologically meaningful outcome. We did this by following the guide by Karen Grace-Martin (https://www.theanalysisfactor.com/sample-size-estimation-without-past-reliable-pilot-data-or-evidence/) and Simon Bates: https://www.nc3rs.org.uk/3rs-resources/how-decide-your-sample-size-when-power-calculation-not-straightforward.
Data exclusions	no exclusion
Replication	all experiments repeated n=3 times (or more). i.e. biological replicates. Data from all replicate experiments was included except where the experiment failed for technical reasons (such as a failed PCR, western blot that didn't transfer etc.).
Randomization	For animal experiments randomization was performed by Mendelian inheritance. For other experiments we did not randomize but used pre-defined conditions for data acquisition so as to avoid any bias.
Blinding	Blinding of mouse experiments was not always possible as the genotype was on the cage card but samples for FACS analysis were barcoded so the genotype of the mouse was not available to the experimenter unless they explicitly looked it up. Similarly for CRISPR screening we made no prior assumptions about what hits would be return from our whole genome screens. Blinding was not possible for other experiments such as transfections.

Reporting for specific materials, systems and methods

We require information from authors about some types of materials, experimental systems and methods used in many studies. Here, indicate whether each material, system or method listed is relevant to your study. If you are not sure if a list item applies to your research, read the appropriate section before selecting a response.

Materials & experimental systems

n/a	Involved in the study	
☐	☒	Antibodies
☐	☒	Eukaryotic cell lines
☒	☐	Palaeontology and archaeology
☐	☒	Animals and other organisms
☒	☐	Human research participants
☒	☐	Clinical data
☒	☐	Dual use research of concern

Methods

n/a	Involved in the study	
☒	☐	ChIP-seq
☐	☒	Flow cytometry
☒	☐	MRI-based neuroimaging

Antibodies

Antibodies used	CD71-FITC antibody (SouthernBiotech, cat. no. 1720-02; 0.5 mg/ml; 1:500)18; SIRT1 antibody (Rabbit Cell Signalling #2496S; 1:1000); anti-centromere antibody (Antibodies Inc, #15-234-0001, 1:1000); anti-rabbit Alexa 488 (ThermoFisher, #A11034, 1:2000); goat anti-human Alexa 647 (ThermoFisher, #A21445, 1:2000). DSCC1 (H0079075-B01P, Novus Biologicals; 1:1000); HSP90 (F-8, Santa Cruz; 1:10.000); HP1-gamma (05-690, Millipore; 1:1000); goat-anti-mouse-PO (DAKO; # P044701; 1:2000). SMC3 antibody (Abcam, AB 9263, 1:250); SMC3 antibody (Thermo Fisher, A300-060A;1:1000,); Anti-acetyl SMC3 mouse antibody (Sigma-Aldrich, #MABE1073, clone 21A7, Lys105/106, LOT: 385016; 1:1000); p53 (Cell Signaling Technology, clone 1C12, #2524S); anti-acetyl p53 (p53-K382Ac; Abcam, ab75754; clone EPR358(2) to p53 acetyl K382; 1:1000); Anti-phospho-Histone H2A.X (Ser139) antibody (clone JBW301;

Sigma-Aldrich,#05-636-I; 1:1000); beta-actin antibody (Merck, #A5441; 1:10000; 5% milk); GAPDH antibody (clone 6C5; Abcam, ab8245; 1:1000); p21 antibody (Abcam, #ab109520; 1:1000).

Validation

The monoclonal CD71-FITC (SouthernBiotech, cat. no. 1720-02) antibody was validated by the manufacturer (and by us) by staining of red cells by flow cytometry and characteristic staining of mouse 18-81 cells.

SIRT1 antibody - we validated this antibody by showing loss of protein expression in a SIRT1 KO line by western blotting.

DSCC1 antibody - validated by the western blotting of DSCC1 KO cell lines (which had been validated by mass-spec).

The anti-centromere antibody (Antibodies Inc, #15-234-0001) was validated by staining of TERT BJ cells (by the manufacturer) and was raised from the serum of a CREST patient. We used the antibody to stain human cells seeing the characteristic staining of centromere.

HSP90 antibody - published >700 times. Validated by knockdown of HSP90 by the manufacturer. https://www.scbt.com/p/hsp-90alpha-beta-antibody-f-8

SMC3 antibody - https://www.thermofisher.com/antibody/product/SMC3-Antibody-Polyclonal/A300-060A - staining, WB

Anti-acetyl SMC3 mouse antibody - https://www.merckmillipore.com/GB/en/product/Anti-acetyl-SMC3-Antibody-Lys105-106-clone-21A7,MM_NF-MABE1073?ReferrerURL=https%3A%2F%2Fwww.google.com%2F . Validated by manufacturer, by IP, WB, staining pattern.

p53 antibody - https://www.cellsignal.com/products/primary-antibodies/p53-1c12-mouse-mab/2524 - WB, staining of cells physiological response. Cited by manufacturer.

anti-acetyl p53 - https://www.abcam.com/products/primary-antibodies/p53-acetyl-k382-antibody-epr3582-ab75754.html . WB: HepG2 cell lysates treated with etopside and TSA. ICC/IF: HeLa cells Flow Cyt (intra): HepG2 cells.

Anti-phospho-Histone H2A.X (Ser139) antibody - https://www.sigmaaldrich.com/GB/en/product/mm/05636?gclid=CjwKCAiAvJarBhA1EiwAGgZl0ETywgkpooS5lhMjBRiEs990yF4OaHxJm8SkY7esNObF8Dfdb4kKoRoCM64QAvD_BwE - physiological response to DNA damage by us and the manufacturer.

beta-actin antibody - https://www.sigmaaldrich.com/GB/en/product/sigma/a5441 - validated by staining of cells, WB, tissue staining.

GAPDH antibody - https://www.abcam.com/products/primary-antibodies/gapdh-antibody-6c5-loading-control-ab8245.html - validated by localisation, size characteristic staining.

p21 antibody - https://www.abcam.com/products/primary-antibodies/p21-antibody-epr362-ab109520.html - validated by KO of p21.

Eukaryotic cell lines

Policy information about cell lines

Cell line source(s)

Mouse embryonic fibroblasts (MEFs) were prepared from E13.5 embryos, following timed matings between Dscc1+/– mice. Briefly, embryos were dissected from the decidium, mechanically disrupted and cultured in DMEM supplemented with 10% fetal bovine serum, 1.0 mML-glutamine, 0.1 mM minimal essential medium nonessential amino acids, 100 µg/ml streptomycin sulfate and 100 U/ml penicillin. The initial plating was defined as passage zero (p0), and cells were subsequently maintained on a standard protocol. SIRT1 KO HEK293 cells were obtained from Kerafast (ENH131-FP). Cells were grown in DMEM, 10% FBS, 1% Penicillin-Streptomycin, 1%GlutaMAX. All other cell lines were from ATCC and STR profiled/validated and certified mycoplasma free. HEK293FT cells were from Thermo Fisher.

Authentication

HEK293 - SIRT1 KO cells - by WB for loss of SIRT1. Previously also confirmed by PCR of the target locus.
HEK293FT - STR profiling (manufacturer). Antibiotic resistance and the ability to package lentiviruses.
iPS cells - validated by whole exome sequencing.
Mouse Fibroblasts - by genotyping of the targeted alleles in embryos.
RPE1 DSCC1 and p53 lines - STR profiling (manufacturer/ATCC) and also PCR of the targeted/mutant alleles.
CHP-212 - STR profiling.

Mycoplasma contamination

All cell lines were mycoplasma tested and found to be free of this contaminant.

Commonly misidentified lines
(See ICLAC register)

No commonly misidentified cell lines were used in the study

Animals and other organisms

Policy information about studies involving animals; ARRIVE guidelines recommended for reporting animal research

Laboratory animals

Mouse; We maintained most of the mice on a pure inbred C57BL/6N background (representing 73% of the mice tested in this study), or for early lines on mixed C57BL/6 backgrounds (e.g., C57BL/6N;C57BL/6BrdTyrc-Brd). For the C57BL/6N background, a core colony was set up using mice from Taconic Biosciences, which was refreshed at set generational points (typically 10 generations) and cryopreserved at regular intervals to avoid genetic drift. All of the ages are provided in the figure legends. With the exception of tumour watch mice all other animals were between 8-15 weeks old.

Wild animals	No wild animals were used in this study.
Field-collected samples	No field collected samples were used in the study.
Ethics oversight	Mouse studies at the Wellcome Sanger Institute (WSI) were performed in accordance with UK Home Office regulations and the UK Animals (Scientific Procedures) Act of 2013 under UK Home Office licenses. These licenses were approved by the WTSI Animal Welfare and Ethical Review Board.

Note that full information on the approval of the study protocol must also be provided in the manuscript.

Flow Cytometry

Plots

Confirm that:

☒ The axis labels state the marker and fluorochrome used (e.g. CD4-FITC).

☒ The axis scales are clearly visible. Include numbers along axes only for bottom left plot of group (a 'group' is an analysis of identical markers).

☒ All plots are contour plots with outliers or pseudocolor plots.

☒ A numerical value for number of cells or percentage (with statistics) is provided.

Methodology

Sample preparation	Briefly, 50 µl of blood was collected from a tail bleed into 300 µl of heparin solution in a 1.5-ml tube and fixed in ice cold methanol at −80 °C in a freezer. Post fixation the samples were washed in bicarbonate buffer and 20 µl of sample was transferred to a 96-deep-well plate (800 µl per well capacity). To each sample 80 µl of CD71-FITC antibody, 7 µl of RNAse and 73 µl of bicarbonate buffer and propidium iodide (PI; 1.0 mg/ml solution Sigma-Aldrich, cat. no. P4864).
Instrument	Samples were analysed on a LSRFortessa or Cytomics FC500, Becton Dickinson.
Software	FlowJo
Cell population abundance	minimum of 100 thousand events for each sample
Gating strategy	we have described in detail the strategy on Nature protocols 10 (1), 205-215. We have also provided a figure in the supplementary Data file.

☒ Tick this box to confirm that a figure exemplifying the gating strategy is provided in the Supplementary Information.
